# Structural Rearrangement of the Olfactory Epithelium in Male Baikal Yellowfin Sculpins Across the Reproductive Period

**DOI:** 10.3390/biology14020179

**Published:** 2025-02-10

**Authors:** Igor V. Klimenkov, Mikhail V. Pastukhov, Hung-Ming Chang, Ting-Yi Renn, Nikolay P. Sudakov

**Affiliations:** 1 Limnological Institute, Siberian Branch, Russian Academy of Sciences, 3 Ulan-Batorskaya St., Irkutsk 664033, Russia; npsudakov@gmail.com; 2 Vinogradov Institute of Geochemistry, Siberian Branch, Russian Academy of Sciences, 1a Favorsky St., Irkutsk 664033, Russia; mpast@igc.irk.ru; 3 Department of Anatomy and Cell Biology, School of Medicine, College of Medicine, Taipei Medical University, Taipei 110301, Taiwan; taiwanose@gmail.com; 4 School of Dentistry, College of Oral Medicine, Taipei Medical University, Taipei 110301, Taiwan; littlenorenn@gmail.com

**Keywords:** olfactory, neuronal plasticity, behavior, fish, reproduction

## Abstract

The morphological peculiarities of receptor neurons and support cells in the olfactory epithelium of male yellowfin sculpin (*Cottocomephorus grewingkii*; Dybowski, 1874) were studied during the pre-spawning, spawning (when males do not feed and have a higher sensitivity to female pheromones), and guarding (the fertilized eggs) periods. This study was performed using electron transmission and laser confocal microscopy. Structural changes in the fish olfactory epithelium are associated with the shift in olfactory signals from alimentary to pheromonal. These results expand our knowledge of the odorant-dependent plasticity of the periphery of the fish olfactory system.

## Simple Summary

The molecular mechanisms of animal behavior directed with smell signals is one of the most enigmatic subjects in modern neurobiology. The most convenient model for research is the reproductive motivation of spawning different fishes when they lose the capability to react on the feed signals, stop feeding, and simultaneously acquire high sensitivity to the sex pheromones. The results of the present work show that, in the spawning period, in the olfactory epithelium cells of Baikal endemic male fish yellowfin sculpin, the ultrastructural rearrangements for adaptation to the reception of sex pheromones of females occur. This is essential for the effective attraction of spawning partners and for successful egg fertilization. The obtained data reveal that the complex behavior of animals is directed not only by central nervous system activity, but also by structural adjustments of the peripheral part of the olfactory analyzer.

## 1. Introduction

Olfaction plays a leading role in organizing the behavior of animals, as it contributes to ensuring their reproduction, nutrition, and social interactions. Currently, the processes associated with the functioning of the olfactory epithelium (OE) are examined both from the point of view of its structural organization and in terms of identifying the regulatory mechanisms that underlie the primary pathways responsible for the perception of olfactory signals. The most important discoveries concerning the functioning of the olfactory epithelium (OE) have been largely achieved in the study of mammals and some other vertebrate species. Molecular biological studies suggest that odor detection thresholds depend on the affinity and the level of expression of olfactory receptors [1,2,3] and on signal amplification and adaptation in the intracellular transduction cascade [4,5,6]. In addition to the type of receptor being expressed, olfactory sensitivity also depends on individual transcriptional profiles in the populations of olfactory sensory neurons (OSNs) that develop in response to specific features of a given environment [7,8]. Scientists are now discussing the possibility of the control of olfactory reception through the participation of purinergic and endocannabinoid systems, whose action actualizes both in the central brain structures and at the OE level [9,10]. Further investigation is needed for the modulation of sensitivity at the OE level through external innervation [11], hormone action, and the involvement of perireceptor processes that change the access, concentration, and chemical structure of odorant molecules [12,13].

In addition to the molecular genetic aspects of olfaction research, it is important to consider issues pertaining to the plasticity of the structural components of the OE in animals undergoing development in the context of a given environment [14]. This also applies to the morphological rearrangements of the OE during periods when chemosensitivity in animals changes substantially due to behavioral rearrangements. To address these issues, we studied the structural and functional rearrangements of fish olfactory epithelium cells during adaptation to spawning, when male fish show increased sensitivity to pheromonal signals from females. At present, the structural and functional peculiarities of fish OSNs are well-characterized. In the OE of fish, the most numerous cells are ciliated and microvillous receptor cells [15,16,17,18,19]. Ciliated OSNs (cOSNs) use a Golf/adenylyl cyclase signaling cascade to activate CNG channels; microvillous OSNs (mOSNs) use a Gq/phospholipase C pathway, together with TRPC2 [20,21,22]. In a previous study, electro-olfactogram recordings were used to show that cOSNs respond to bile salts and that mOSNs are sensitive to amino acids [23]. Similar responses of microvillous neurons to amino acids were also recorded in [20,24]. Based on a study of olfaction in rainbow trout, Sato and Suzuki (2001) argued that cOSNs are “generalists”, i.e., that they respond to a wide range of odors, including pheromones, while mOSNs are “specialists”, specific to amino acids [25]. Hansen et al. (2003) conducted a study in channel catfish to show that microvillous neurons can respond to nucleotides and that amino acid odorants activate both ciliated and microvillous neurons, but via different signaling pathways [21]. The third type of receptor elements in the OE of fish includes crypt cells [26]. They have been shown to express the G proteins Gαo and Gαq, adenylate cyclase III, and the glial marker proteins S-100 and TrkA. Crypt cells were shown to express a single V1R receptor, i.e., V1R4, coupled to Gαi; although their ligands are unknown, it was suggested that these receptors respond to pheromones [27]. Cytochemical studies in crucian carp have shown that the localization of pheromone-sensitive crypt cells varies substantially throughout the year; in summer, i.e., during the transition to spawning, their bodies move to more superficial layers of the epithelium [28]. Recently, the patch clamp method and intravital Ca^2+^ ion imaging were used in a study on mackerel and juvenile trout to show that different subpopulations of crypt cells respond to amino acids, bile acids, or pheromonal signals [29,30].

In the course of studying the olfactory apparatus of zebrafish, other “crypt-like” cells were also identified in the OE, which sent their axons to a glomerulus that was different from other cell types [27,31]. It turned out that these unusual cells, which were called kappe neurons because of their characteristic shape, expressed Gα s/olf proteins and produced no specific markers typical of ciliated, microvillous, or crypt cells. Immunochemical staining of the kappe cells revealed no tubulin in them, leading some authors to believe that they did not contain cilia [27].

Recently, another small population of OSNs, pear-shaped neurons, was described in the surface layers of the OE in zebrafish [32]. These neurons were shown to express the A2c receptor, which is present in lower aquatic organisms and mediates the recognition of adenosine [33].

Despite the well-known data on the structural and functional specialization of different OSN types, the structural plasticity of those cells at different stages of fish reproductive maturation (associated with the shift in the dominating odorant) has been poorly studied. In this work, we examined fish endemic to Lake Baikal (yellowfin sculpin, *Cottocomephorus grewingkii* Dybowski, 1874) (Cottidae) during the key stages in their reproductive cycle, when (based on the results of behavioral experiments) their odorant perception spectrum is reconfigured from food to sex pheromonal signals [34,35,36]. As the water of Lake Baikal is very pure, it can be used as a unique natural test site to collect basic data, which is a challenge in studies of the sensory systems of aquatic organisms from other, often more polluted, bodies of water. With electron and laser confocal microscopy, we studied the cytochemical rearrangements of the receptor and support cells of the olfactory epithelium of male *C. grewingkii* 1 month before reproduction, during spawning, and after 1 month of parental behavior (guarding fertilized eggs). For the first time, the transition of ciliated OSNs to a dendritic neurosecretion mode was revealed and characterized. In this mode, the activated Golgi apparatus produces a higher number of vesicles, which are transported to the apical side of the cell, are further incorporated into the plasmalemma, and release their content into the olfactory mucus. The obtained data are very important for understanding the animal’s OE adaptive rearrangements in the context of their behavioral changes.

## 2. Materials and Methods

### 2.1. A Natural Model of Changes in Olfactory Sensitivity in Fish

**Figure 1:**
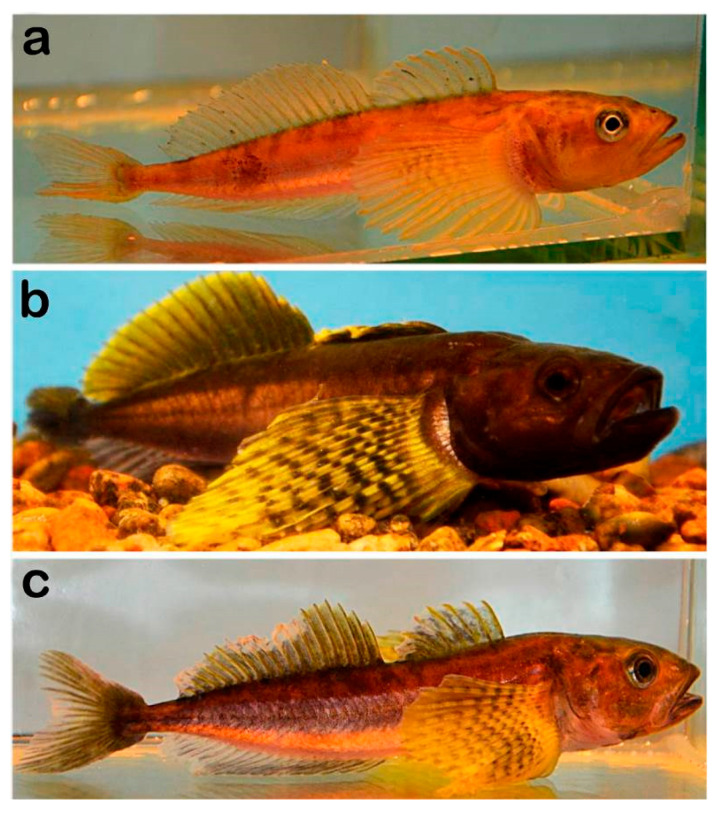
Male yellowfin (*Cottocomephorus grewingkii* (Dybowski, 1874; Cottidae) in the pre-spawning ((**a**); stages 3–4 of maturity), spawning ((**b**); stage 5 of maturity) and parental behavior period (**c**).

To study the adaptive rearrangements in the olfactory apparatus of fish at different stages of their life cycle and spawning behavior, males of the sculpin *Cottocomephorus grewingkii* (Dybowski, 1874) (Cottidae) an endemic representative of the ichthyofauna of Lake Baikal, were selected. The main criterion for selecting the research object was the fact that olfactory sensitivity in male fish switches from the perception of food signals to female sex pheromones during the spawning period, and then shuts down when male fish switch to parental behavior (spawn protection). The yellowfin (*C. grewingkii*) is a benthopelagic species. During the interspawning season (stages 3–4 of maturity) (Figure 1a), the sculpin lives at a depth of 70–200 m [37]. At this time, its diet consists mainly of zooplankton, primarily the copepod Epischura baicalensis Sars 1900 and the pelagic amphipod *Macrohectopus branickii* (Dybowsky, 1874). When approaching the spawning ground in the coastal zone of the lake, the males stop feeding and acquire bright mating coloration (stage 5 of maturity) (Figure 1b). Their reproductive behavior includes searching for a nest, attracting a female [35], and fertilizing the eggs of 6–10 females, which successively arrive at the nest.

Behavioral tests have revealed that, during the transition to reproduction, males exhibit highly differentiated sensitivity to the female primer pheromone at a concentration of 10^−11^–10^−13^ M [34,36]. At the end of spawning, males cease being olfactorily sensitive to female pheromones and start to protect the eggs, which lasts for 35–40 days until the larvae hatch [37,38]. During this period, male gobies are mostly in the nest. They lose spawning dress and do not feed [39] (Figure 1c). Based on behavioral experiments, we can say that the radical reconfiguration of olfaction in fish from food signals to pheromonal ones to ensure spawning, followed by the weakening of olfaction during the transition to the protection of eggs, makes it possible to reveal the corresponding structural rearrangements in OE cells.

All fish were caught using fixed nets in February–March, May, and August–October 1982–2022 in South Baikal. Morphological material (olfactory rosettes) was taken from the hydrobionts in the pre-spawning period (stages 3–4 of maturity) at a depth of 100 m (*n* = 25), during spawning (stage 5 of maturity) in the coastal zone of the lake at the sites of their natural spawning grounds (*n* = 50), and in the transition phase where males protect fertilized eggs (*n* = 6). The maturity stage of the fish was determined using the gonadosomatic index [40]. In our work, we used yellowfin males (*Cottocomephorus grewingkii* (Dybowski, 1874; Cottidae) in the pre-spawning (*n* = 50; length total 116.2 ± 2.06 mm; weight 17.3 ± 1.26 g; age 2+ years), spawning (*n* = 50; length total 119.8 ± 1.43 mm; weight 21.6 ± 2.18 g; age 3 years), and post-spawning periods (*n* = 6; length total 118.6 ± 1.24 mm; weight 13.6 ± 1.03 g; age 3 years).

### 2.2. Autopsy

The manipulations for olfactory rosettes excision were performed by using a “Biomed” MS-1 stereomicroscope. For olfactory rosette preparation, the skin with underlaying tissues between input and output holes of olfactory cavity was removed with forceps and scalpel. Then, using a scalpel and ophthalmic lancet, the surrounding connective tissue layer was removed, and subsequently, the emerging olfactory nerves were cut and the olfactory rosettes extracted. Caught fishes were subjected to euthanasia with tricaine mesylate (MS222) [41], according to the protocol of the AVMA Guidelines for the Euthanasia of Animals: 2020 Edition. We used the recommended dosage of MS222 for the euthanasia of the fish: 250 mg/L. After extraction, the olfactory rosettes were rinsed in 0.1 M phosphate-buffered saline (PBS, pH = 7.3) and immersed in a fixative or stain solution depending on the microscopy method.

### 2.3. Microscopic Investigation

We used transmission, scanning electron, and confocal microscopy to study the structural peculiarities of OSNs and SCs in the fish.

#### 2.3.1. Transmission Electron Microscopy

Olfactory rosettes (*n* = 40) were fixed in 2.5% glutaraldehyde (Merck, Darmstadt, Germany, Cat. No. G5882) solution in PBS at 4 °C for 2 h. After washing with PBS, biomaterial was postfixed in 2% OsO_4_ (Merck, Darmstadt, Germany) in the same buffer for 12 h, washed in PBS, and dehydrated in an ascending alcohol gradient with acetone. The dehydrated specimens were embedded in Araldite 502 resin with a 2,4,6-tris dimethylamino methylphenol (DMP-30) catalyst (Araldite 502 Kit, SPI Supplies, West Chester, PA, USA). Ultrathin sections (70–80 nm) were made with an Ultracut R microtome (Leica Microsystems, Wetzlar, Germany), placed on copper grids, and contrasted in lead citrate according to Reynolds (1963) [42]. The grids prepared with specimens were examined under a Leo 906E transmission electron microscope. Images were captured with a Mega View II digital camera and processed using the Mega Vision soft package (Soft Imaging System GmbH, Muenster, Germany). The comparative analysis of ribosomes quantity in the OSNs cytoplasm in fishes on the different reproductive stages was performed indirectly by evaluating the optical density with Image Pro+. On the whole, we analyzed 60 images for each group (pre-spawning and spawning) fish. We evaluated the mean length between nuclear pores in pre-spawning and spawning fish with Image Pro+ 6.0.0.260 soft (Media Cybernetics, Inc., Rockville, MD, USA). Based on these data, we calculated the number of pores per 1 µm^2^. On the whole, we analyzed 40 images for each group.

#### 2.3.2. Scanning Electron Microscopy

Olfactory rosettes (*n* = 35) were fixed in 2.5% glutaraldehyde, washed in PBS, postfixed in 2% OsO_4_, and dehydrated in an ascending alcohol gradient. The prepared olfactory rosettes were mounted on SEM specimen stubs, dried at a critical point with a Bal-Tec Critical Point Dryer CPD 030 (Balzers, Sercolab, Merksem, Belgium), and coated with gold with an SCD 004 sputter coater (Balzers, Sercolab, Merksem, Belgium). The specimens were analyzed with a scanning electron microscope, FEI Quanta 200 (FEI Company, Hillsboro, OR, USA).

#### 2.3.3. Laser Confocal Microscopy

For the synchronous visualization of SC apical areas with functionally active mitochondria, we stained the olfactory rosettes with MitoTracker^®^ Orange CMTMRos (Thermo Fisher Scientific Inc., Waltham, MA, USA, Cat. No. M7510) and phalloidin–FITC (Thermo Fisher Scientific Inc., Waltham, MA, USA, Cat. No. F432) [43].

*Functionally active mitochondria*. Olfactory rosettes were incubated with 25 nM MitoTracker^®^ Orange CMTMRos (Thermo Fisher Scientific Inc., Waltham, MA, USA, Cat. No. M7510) and dissolved in medium 199 (Pan Eco Ltd., Moscow, Russian Federation, Cat. No. C230p) for 30 min for functionally active mitochondria staining [44]. The stained rosettes were fixed with 4% paraformaldehyde for 15 min and stained with DAPI (Merck, Darmstadt, Germany, Cat. N D9542) for 15 min. After each step, the prepared specimens were washed three times in PBS. The prepared olfactory rosettes were embedded in ProLong Gold Antifade Mountant (Thermo Fisher Scientific, Waltham, MA, USA, Cat. No. P36930).

*Actin microfilaments (F-actin)* were stained with phalloidin–FITC (Thermo Fisher Scientific, Waltham, MA, USA, Cat. No. F432) [43]. For this purpose, olfactory rosettes were fixed in a 4% paraformaldehyde (Merck, Germany, Cat. No. P6148) solution in PBS for 15 min. After fixation, the specimens were permeabilized with 0.25% Triton X100 (AppliChem GmbH, Darmstadt, Germany, Cat. No. 142314) solution in PBS for 30 min. The olfactory rosettes were stained with 165 nM phalloidin–FITC for 40 min and with 10 µg/mL DAPI (Merck, Germany, Cat. N D9542) for 15 min. Washing with PBS (×3 fold) was conducted after each step of specimen preparation. The stained olfactory rosettes were placed on a slide, embedded in ProLong Gold Antifade Mountant (Thermo Fisher Scientific Inc., Waltham, MA, USA, Cat. No. P36930), and sealed with a coverslip.

*Microtubules*. For dendritic microtubule (important components of OSN development and functioning [45,46]) staining, we used the antibodies against α-tubulin conjugated with Alexa488. Olfactory rosettes were fixed with 4% paraformaldehyde with 0.25% Triton X100 and stained with diluted 1:100 Alexa Fluor 488 Anti-α tubulin antibody [DM1A] (Abcam plc, Cambridge, UK, Cat. No. ab195887) for 1 h (37 °C). After tubulin staining, the specimens were stained with 2 µg/mL Hoechst 33342 (Thermo Fisher Scientific Inc., Waltham, MA, USA, Cat. No. H3570). After each step of specimen preparation, we washed the olfactory rosettes with PBS three times. Ready-made samples were embedded in ProLong Gold Antifade Mountant (Thermo Fisher Scientific Inc., Waltham, MA, USA, Cat. No. P36930).

*Laser confocal microscopy image analysis*. The processing of the 3D scans and the extraction of images were carried out with a Zen 2010 (Carl Zeiss, Oberkochen, Germany), Imaris Viewer 9.6.0, and an Imaris Bitplane 7.2.3 (Bitplane AG, Belfast, UK). The status of the mitochondria functional activity in the olfactory rosettes was estimated as the volume of functionally active mitochondria in 1 × 10^6^ µm^3^ of tissue. To analyze the dendritic microtubules, functionally active mitochondria, and actin microfilaments, we obtained 15–20 Z-stacks from each olfactory rosette. We used 9 olfactory rosettes from pre-spawning and spawning fish. For microtubule staining, we also used 9 preparates each. The average thickness of the Z-stacks was 20–30 μm. We examined the olfactory rosettes layer by layer using 2D sections or bulk Z-stacks or by making orthogonal projections of selected areas.

### 2.4. Statistical Analysis

We analyzed the data using non-parametric statistics, and we calculated the median and quartiles with the Statistica 10 software package (Stat Soft Inc., Tulsa, OK, USA). Intergroup differences were estimated using the Mann–Whitney U test. Intergroup differences were considered significant at *p* ≤ 0.05.

## 3. Results

### 3.1. Morphological Features of OE Cells in Male Fish in the Pre-Spawning Period

**Figure 2:**
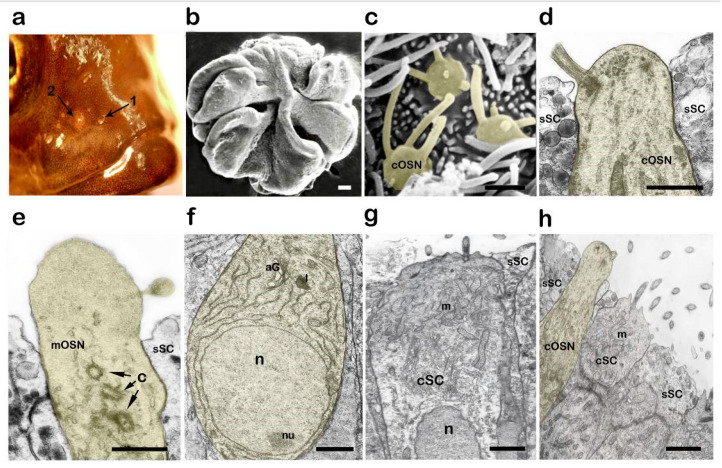
Structural features of the olfactory epithelium in the male *C. grewingkii* in the pre-spawning period. (**b**,**c**) Scanning electron microscopy. (**d**–**h**) Transmission electron microscopy. (**a**) The olfactory rosette occupies an anterodorsal position in the head; the nasal cavity has an inlet (1) and an outlet (2). (**b**) General view of the olfactory rosette. (**c**,**d**) Ciliated OSN. (**e**) Microvillous OSN; the centrioles (C) are marked with arrows. (**f**) OSN body with endoplasmic reticulum channels and Golgi apparatus; the nucleolus is located at the lower pole of the nucleus. (**g**) Section of a ciliated supporting cell with a small number of mitochondria located at the base of the cilia. (**h**) Apical sections of cOSN, cSC, and sSC. Different areas of the receptor cells are artificially colored yellow. Notation: cOSN—ciliated olfactory sensory neuron; mOSN—microvillous olfactory sensory neuron; cSC—ciliated supporting cell; sSC—secretory supporting cell; aG—Golgi apparatus; m—mitochondria; n—nucleus; nu—nucleolus; l—lipofuscin. Scale bar: (**b**) 100 µm; (**c**–**h**) 1 µm.

The peripheral part of the olfactory system is located on both sides of the dorsal part of the head and is represented by paired organs, i.e., rosettes, which are located at the bottom of special pits. Each of the pits is covered from the outside with a skin fold, which has two openings: an inlet and an outlet (Figure 2a). These openings serve to connect the nasal sac with the external environment and enable a continuous flow of water to be tested in the olfactory chamber. In adult fish, the rosette is 1.5–2 mm in diameter and consists of 5–6 folds extending from the central septum (Figure 2b). The olfactory epithelium overlays both sides of each lamellae, except for the end sections, which have nonciliated epithelial cells, among which mucous goblet cells occur. The thickness of the sensory epithelium is, on average, 45–50 µm. Like other bony fish, the OE in Baikal Cottoidei has a structure that is typical of vertebrates and consists of receptor, supporting, and basal cells. The olfactory lamellae of Baikal sculpins have no secondary folding, with the receptor and supporting elements being distributed in the OE in a mixed order without forming isolated zones.

The OE of *C. grewingkii* is observed to contain ciliated (Figure 2c,d) and microvillar OSNs. (Figure 2e). The apical section of the cOSN ends in an olfactory knob with four to six cilia. The cilia are 4–6 µm in length. The apex of the mOSNs, which are rare in the epithelium, contains a few digitules (1–3 μm), which have neither basal bodies nor a microtubular apparatus. According to the observations, there is usually 1 microvillar cell per 8–10 ciliated receptor cells. It is revealed that frequency of occurrence of OSNs on the surface of lamella is 11 (10; 13) cells per 100 μm^2^ of epithelium.

The OSN bodies form several rows in the epithelium thickness. The bodies have a fusiform shape, with the nucleus-containing zone of the cells usually being 3–4.5 µm in diameter (Figure 2f). The weakly expressed nucleolus is located in either the upper or lower pole of the nucleus. The perinuclear zone of the cells is filled, to a degree, with a wide network of channels of the granular endoplasmic reticulum. Individual mitochondria, lysosomes, multivesicular bodies, and a few ribosome rosettes are localized in the cytoplasm.

The Golgi apparatus normally has low secretory activity. Near the trans-disks, the newly formed vacuoles break up, as a rule, into smaller (0.1–0.2 μm) vesicles (Figure 2f). A characteristic feature of OSNs in fish in the pre-spawning period is the presence of granules (likely of a lipofuscin nature) in their cytoplasm. The granules usually have an irregular shape and an increased electron density. A large amount of lipofuscin is a characteristic feature of OE cells in other Baikal Cottoidei [47].

Like other vertebrates, two types of supporting cells—ciliated and secretory—are identified in the OE of the sculpins. The ciliated cells are rectangular in shape with a wide flat apex with 9–16 extending cilia (Figure 2g). Mitochondria tend to concentrate in the apical part of the cell. The cytoplasm in the circumnuclear zone has a low electron density and contains few organelles. Apart from the Golgi apparatus, here, one can localize short fragments of channels of the granular endoplasmic reticulum and a few free ribosomes.

The secretory supporting cells are located between the bodies of the receptor and ciliated cells, and they are largely characterized by weak secretory activity (Figure 2h).

### 3.2. Structural Rearrangements of OE Cells in the Spawning Period

**Figure 3:**
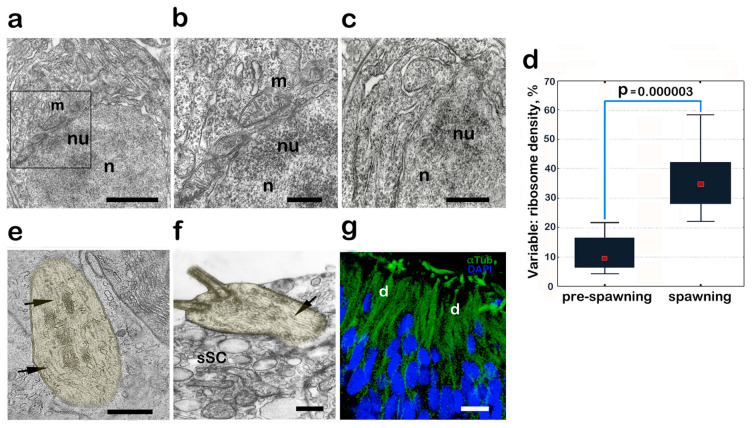
Structural rearrangements of OE cells in male *C. grewingkii* during nest search and spawning behavior. (**a**–**c**,**e**,**f**) Transmission electron microscopy; (**g**) confocal microscopy. (**a**) Mitochondria are located close to the nuclear membrane; the nucleolus is located in the upper pole of the nucleus. (**b**) Enlarged fragment (**a**). (**c**) A large number of ribosomes in the cytoplasm and on the channels of the endoplasmic reticulum. (**d**) The graph shows an increase of 3.6 times (*p* = 0. 000003) in the optical density of ribosomes in olfactory receptor cells during the transition of male yellowfin from the pre-spawning period (1) to the spawning (2) period. (**e**) Parallel oriented microtubules in OSN dendrite (artificially colored yellow) (arrow–microtubules). (**f**) Apex cOSN with microtubules (marked by arrow). (**g**) Staining for microtubules (antibodies to α-tubulin, green) and nuclei (DAPI, blue) in OSNs. Different areas of the receptor cells are artificially colored yellow. Notation: n—nucleus; nu—nucleolus; d—OSN dendrite; sSC—secretory supporting cell; m—mitochondria. Scale bar: (**a**,**c**) 1 µm; (**b**,**d**,**e**) 0,5 µm; (**f**) 10 µm.

When male fish are in the period of nest selection and the formation of sexual behavior, their OE cells undergo well-pronounced ultrastructural rearrangements, with the aim of increasing their functional status. The OSNs show signs of activation of nuclear–cytoplasmic interactions. In many OSNs, the nucleolus shifts to the upper pole of the nucleus and acquires a well-defined granular component. Compared with the previous period, the number of nuclear pores per unit area increases by a factor of 1.4–1.7 (from 9 to 12–15 pores per 1 µm^2^; *p* ≤ 0.01). The outer nuclear membrane is observed to have an increased number of ribosomes, which form regular groups. In the immediate vicinity of the pores and in the adjacent zones, there are localized mitochondria with well-defined cristae (Figure 3a,b). They lie tightly adjacent to the nuclear membrane in the upper pole of the nucleus, close to the nucleolar area.

The activation of the OSNs’ functional status is also reflected in the increased number of both free and reticulum channel-related ribosomes. As a rule, they are pooled into polyribosomal complexes, which occupy considerable cytoplasmic space in the supranuclear region and, partially, under the nucleus (Figure 3c). The comparison of optical density data of the cytoplasm with ribosomes in OSNs of pre-spawning and spawning fish shows a 3.6-fold increase of this parameter by (*p* = 0.000003) during reproduction (Figure 3d). In the OSN dendrites, one can observe an increased number of mitochondria and microtubules, which begin in the cell body and end in the olfactory knob matrix (Figure 3e,f). In the apical part of the dendrites and inside the olfactory knob, the microtubules exhibit an especially high level of ordering—they line up parallel to each other, ending near the basal bodies of the cilia. This is also evidenced by the confocal microscopy data obtained after the α-tubulin staining of cell dendrites (Figure 3g).

**Figure 4:**
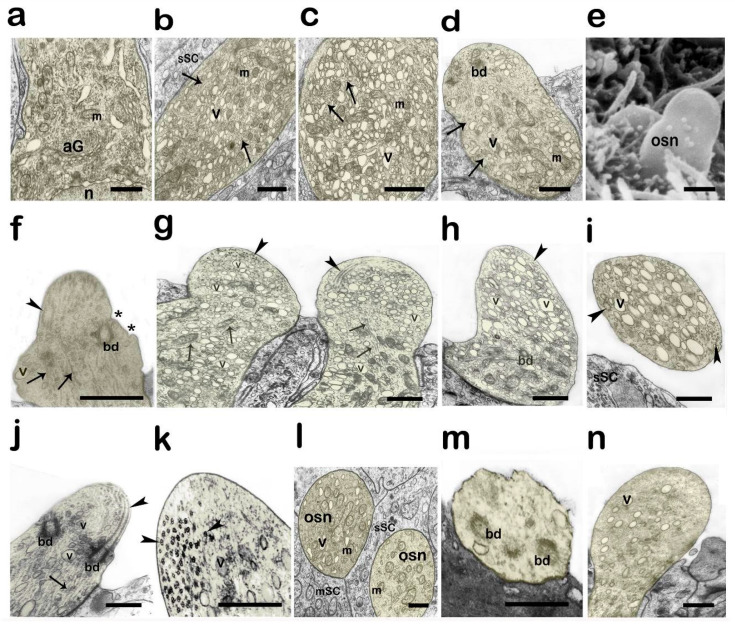
Cytological features of OSN transformation to dendritic neurosecretion in male *C. grewingkii* during the transition to parental behavior. (**a**–**d**,**f**–**l**) Transmission electron microscopy; (**e**) scanning electron microscopy. (**a**) Subnuclear zone of OSN with signs of active protein synthesis; a large number of ribosomes and a well-defined Golgi apparatus. (**b**) OSN dendrite with a large number of mitochondria, microtubules, and secretory vesicles; arrows indicate dendritic microtubules. (**c**) Transverse section of an OSN dendrite showing many mitochondria and secretory vesicles. (**d**) Apex of OSN without cilia; mitochondria and secretory vesicles are visible among the microtubules. (**e**) Modified shape of the olfactory knob without cilia. (**f**) Structural transformation of the olfactory knob during dendritic neurosecretion; sites of incorporation of secretory vesicles in the surface membrane are marked with an asterisk. (**g**) The apical parts of two closely located cOSNs with a high number of secretory vesicles. (**h**) Highly elongated olfactory knob with vesicles. (**i**) Transverse section through a modified olfactory knob with a large number of secretory vesicles. (**j**) Transverse section through a modified olfactory knob with a large number of secretory vesicles. (**h**) Olfactory knob with preserved ciliary axoneme located under the surface membrane of the cell; numerous vesicles and microtubules extending in the dendrite. (**k**) Degenerative changes in the olfactory knob; disaggregation of microtubules of cilia (indicated by arrows), a small number of vesicles. (**l**) Two adjacent profiles of dendrites OSNs, in one of which secretory vesicles are observed. (**m**) Apex of a cOSN in the process of degeneration. (**n**) Apical region of a microvillous OSN with secretory vesicles. Different areas of the receptor cells are artificially colored yellow. Notation: cSC—ciliated supporting cell; sSC—secretory supporting cell; aG—Golgi apparatus; m—mitochondria; n—nucleus; v—vesicle; bd—basal body; small arrow—dendrite microtubules; big arrow—cilia microtubules. Scale bar: 1 µm.

Against the background of increased protein synthesis in individual cells, one can observe the fragmentation of the endoplasmic reticulum and the formation of small vacuolar structures. In addition, many (15–20%) OSNs demonstrate ultrastructural rearrangements characteristic of being in the state of dendritic neurosecretion. In such cells, numerous vesicles with a diameter of 0.1–0.5 μm with light content can be seen near the transdisks’ Golgi apparatus (Figure 4a). Similar vesicles are also located between the mitochondria and the microtubules throughout the entire length of the dendrite, and they then penetrate into the olfactory knob. Stretching along the dendrite, the microtubules bend at different angles, as a result of which the sections show bundles of microtubules, in both the transverse and longitudinal sections (Figure 4b).

The dendrite diameter in this period is usually greater, by a factor of 1.5–3, than the typical one in the pre-spawning period (Figure 4c). Both the sections (Figure 4d) and scanning microscopy (Figure 4e) show that OSNs with a vigorous synthesis of vesicles, transported along the dendrite, have a knob without cilia (or microvilli). How can the cells lose their superficial chemosensitive apparatus? The cuts show that, as the vesicles enter the knob matrix, they come close to the surface membrane, become incorporated into it, and release their contents into the olfactory mucus (Figure 4f). As the vesicle membrane material becomes incorporated into the surface membrane, its volume gradually increases. The membrane becomes deformed, stretching out to a length that may be sometimes twice to four times greater than the original one (Figure 4g). This growing zone shows the presence of a large quantity of vesicles, which are continuously delivered from the cell body. In terms of morphological features, the modified knob resembles, in many respects, the anterior segment of a growing axon, i.e., the growth cone [48,49]. Thus, one of the main goals was to study the structural changes in the dendrite terminal during which the cell loses its cilia (Figure 4d–g). It is known that olfactory cilia undergo destructive changes during natural cell death [50] when exposed to detrimental physicochemical factors [51] and in the case of ciliopathies [52].

It was found that, during the transformation of OSNs into the dendritic neurosecretion mode, the cilia are not shed outward, as is the case after exposure to surfactants [51]. The cilia are detected in the olfactory knob matrix, with the axoneme microtubules initially retaining their spatial organization ((9 × 2) + 2), which is characteristic of eukaryotes [53] (Figure 4h). Afterwards, when immersed inside the dendrite, the cilia gradually disaggregate. Such cells are found to also have, along with intact axonemes, ones in which the principle of strict mutual arrangement is noticeably violated. In this case, the structural bonds between the doublets of neighboring microtubules are damaged, which leads to their mutual displacement and the destruction of the original native architectonics (Figure 4i). Importantly, in this case, the microtubules are still arranged in pairs, suggesting high structural stability. It is characteristic that vesicle formation does not occur in all the olfactory cells. Figure 4j shows the cross-sections of two adjacent dendrites of OSNs surrounded by SC bodies. These sections clearly demonstrate their different structural development, although the cells are spaced at a small distance apart. The profile of one of the projections shows organelles typical of this sensory cell’s zone, i.e., mitochondria and elements of the smooth endoplasmic reticulum, whereas the analogous area of the neighboring neuron contains numerous secretory vesicles. As mentioned above, the activation of the synthesis of secretory vesicles occurs against the background of enhanced protein synthesis and leads to dendritic neurosecretion from the apical part of the cell. What is the potential duration of the secretory process that arises in the olfactory cell? Is the cell able to return to its original state by assuming its previous structural organization, including the apical section? In our preparations, among the receptor cell apices filled with vacuoles, we observed dendritic terminals of the same shape yet with a cleared matrix, in which we could distinguish fragments of destroyed basal bodies (Figure 4k). We did not obtain decisive confirmation as to which receptor cells these knobs belonged to—the cells depleted as a result of a secretory process occurring in them, or typical cells degenerated at the end of their life cycle. It was also unclear whether the central projections were preserved in the cells whose receptor region lost its chemosensitive elements in the course of the morphological rearrangements.

**Figure 5:**
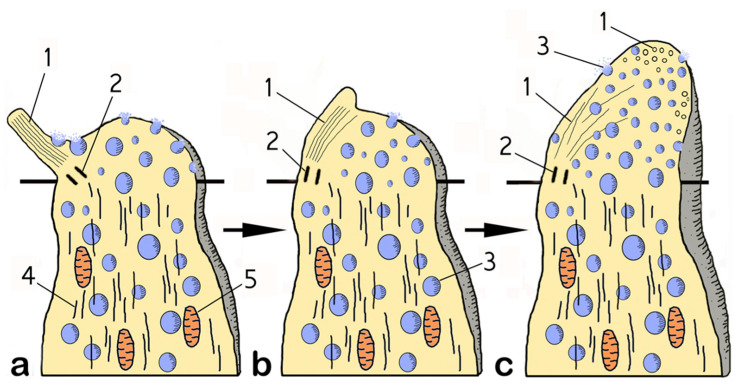
Generalized scheme of structural reorganization of the tip of a cOSN when it switches to the dendritic neurosecretion mode in male *C. grewingkii*. (**a**) Transport of vesicles to the upper part of the cell and their incorporation into the surface membrane. The cilia has the usual location. (**b**) The axoneme of the cilia is immersed in the matrix of the olfactory knob. (**c**) Growth olfactory knob due to the embedding of vesicles with the cell surface membrane; disaggregation of the axoneme of the cilia. Notation: 1—cilia microtubules; 2—basal body; 3—vesicle; 4—cytoplasmic microtubules; 5—mitochondria.

**Figure 6:**
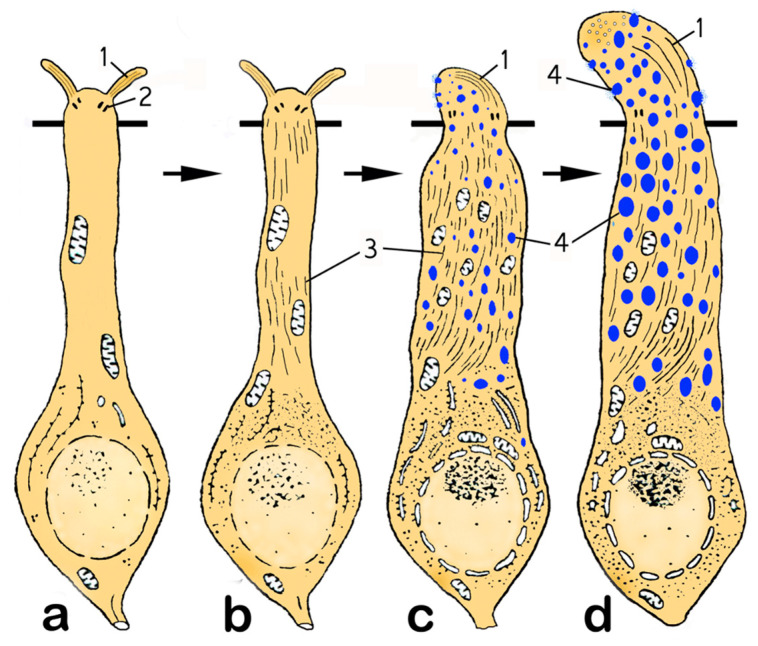
Ultrastructural features of cOSNs in the pre-spawning period (stages 3–4 of maturity) (**a**) and in different phases of reproductive behavior (stage 5 of maturity) (**b**–**d**) in male *C. grewingkii*. (**b**) Period of maturation and release of reproductive products. (**c**,**d**) Phases of differentiation of olfactory cells into the mode of dendritic secretion during the period of completion of spawning and transition to parental behavior. Notation: (1) cilia; (2) basal body; (3) dendrite microtubules; (4) secretory vesicles.

A similar increase in the volume of the apical region due to the incorporation of vesicles was also observed in the rare (for every 8–10 ciliary OSNs, there is 1 microvillary OSN) microvillar receptor cells. Figure 4l shows a microvillar cell with light-colored vacuoles entering the olfactory knob. Thus, the most significant structural rearrangements of the vertices of OSNs in fish during spawning are observed in ciliated OSNs, and they are presented in Figure 5. A general scheme of the ultrastructural rearrangements of ciliated OSNs in the pre-spawning period and in different phases of reproductive behavior in male *C. grewingkii* is shown in Figure 6.

**Figure 7:**
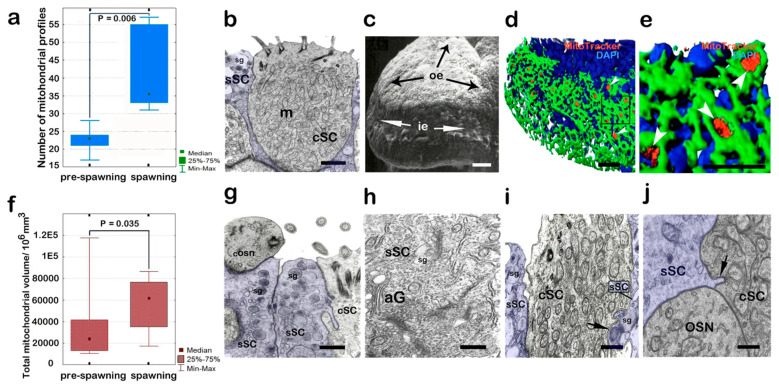
Structural features of supporting cells of the olfactory epithelium in male *C. grewingkii* during the spawning period. (**a**,**g**–**j**) Transmission electron microscopy; (**c**) scanning microscopy; (**d**,**e**) confocal microscopy. (**a**) Ciliated supporting cell with many mitochondria. (**b**) The graph shows a 1.5-fold increase (*p* = 0.006) in the number of mitochondrial profiles in the ciliated supporting cells of the olfactory epithelium during the transition of the male yellowfin from the pre-spawning period to the spawning; according to transmission electron microscopy data. (**c**) The surface of the lamellae of the olfactory rosette; receptor epithelium (re); indifferent epithelium (ie). (**d**) Fragment of the lamellae of the olfactory rosette, similar to the drawing (**c**); staining for F-actin (FITC phalloidin, green), for mitochondria (MitoTracker^®^Orange CMTMRos, red) and for nuclei (DAPI, blue); white arrows show the tops of cSC with mitochondria. (**e**) Enlarged fragment (**d**). (**f**) The volume of functionally active mitochondria in OE during the pre-spawning period and during spawning; the graph shows the data obtained through the quantitative analysis of the Z-stacks; confocal microscopy. (**g**) The apical part of the secretory SC with secretory granules. (**h**) Secretory supporting cell cytoplasm with active Golgi apparatus. (**i**) Invagination of the cytoplasm with secretory granules (indicated by arrow) from the side of the sSC into the cSC in the spawning period. (**j**) Invagination of the cytoplasm (indicated by an arrow) from the side of the sSC into the cSC in the pre-spawning period. Different areas of the secretory SC are artificially colored blue. Notation: cSC—ciliated supporting cell; sSC—secretory supporting cell; aG—Golgi apparatus; m—mitochondria; sg—secretory granules. Scale bar: (**a**,**g**–**j**) 1 µm; (**d**–**e**) 20 µm; (**c**) 10 µm.

When the fish transition to the spawning phase, ultrastructural changes are observed not only in the OSNs, but also in the OE supporting cells (SCs). In particular, compared with the pre-spawning period, the number of mitochondria in the ciliated SCs at the base of the cilia’s basal bodies increases by 1.5 times (*p* = 0.006) (Figure 7a,b). This pattern is also confirmed by confocal microscopy with the simultaneous cytochemical staining of both mitochondria and actin microfilaments in the cingulae of cell apices. We previously showed that, when phalloidin-FITC preparations are treated, the profiles of supporting elements look wider than those of receptor cells and are easily detected [54]. Figure 7c depicts a separate fold of the yellowfin’s olfactory rosette, where the surface of the OE is clearly visible, bordering on the indifferent epithelium. Figure 7d depicts a similar section of the olfactory rosette, stained for actin microfilaments and functionally active mitochondria. Figure 7d,e show that the apical regions of ciliated SCs with a well-pronounced fluorescent signal from mitochondria are dispersed with a high level of ordering in the olfactory fold. A statistical analysis of the entire pool of mitochondria (including the mitochondria of receptor cells and other types of cells) detected in the OE in the reproduction phase shows that the indicators in the previous pre-spawning period are exceeded by a factor of 2.3 (*p* ≤ 0.05) (Figure 7f). This indicates an increased level of energy metabolism in the sensory apparatus of the fish during this period. Moreover, signs of a substantial increase in secretory activity are observed during this period in supporting mucous cells (Figure 7g). In the perinuclear zone of cells, one can clearly identify evenly expanded channels of the granular endoplasmic reticulum with numerous ribosome rosettes and the Golgi apparatus, with vacuoles of various sizes (0.2–0.8 μm) and electron densities localized near the latter (Figure 7h). Moreover, in some cases, vesicles within the deep cytoplasmic projections of secretory SCs penetrate into the cytoplasm of neighboring ciliated cells (Figure 7i). It is possible that these protrusions, together with their contents, can be engulfed, and, in this way, the ciliated cells assimilate the substances formed in the mucous cells. Such sprouts between these cells are also observed during the pre-spawning period (Figure 7j).

### 3.3. Structural Features in the OE During Parental Behavior

**Figure 8:**
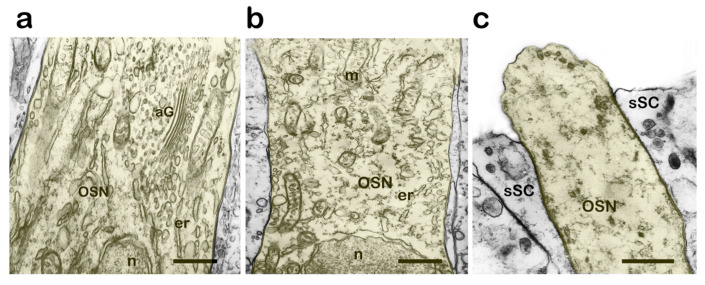
Degenerative changes in the cells of the olfactory epithelium a month after the protection of fertilized eggs in male *C. grewingkii*; transmission electron microscopy. (**a**,**b**) Nuclear zone with fragmented channels of the endoplasmic reticulum in OSN. (**c**) Apical section of the dendrite OSN with an enlightened cytoplasmic matrix; cilia, mitochondria, and microtubules are missing. Notation: OSN—olfactory sensor neuron; sSC—secretory supporting cell; aG—Golgi apparatus; m—mitochondria; n—nucleus; er—endoplasmic reticulum. Scale bar: 1 µm.

A month after the beginning of the protection of fertilized eggs, neurodegenerative changes are observed in OSNs and other OE cells, which are accompanied by the clearing of the cytoplasmic matrix and the destruction and fragmentation of the main structural components of both the cell body (Figure 8a,b) and its apical regions (Figure 8c). Similar changes are observed in supporting cells: the cell cytoplasm is depleted in intracellular components; the secretory activity of the Golgi apparatus does not occur; and the disks stay as close as possible to each other and produce virtually no vesicles. During this period, the fish basically stop feeding, do not respond to sex pheromones, and keep guarding the eggs until they hatch.

## 4. Discussion

We studied the structural features of the olfactory epithelium (OE) in male yellowfin sculpins at different stages in their life cycle: in the prespawning period, during spawning, and during the transition to guarding the fertilized eggs.

Like in the other previously studied representatives of *Cottoidei* [44,47,54], the OE of yellowfins was found to contain ciliated and microvillous OSNs. Crypt cells confirmed to be involved in the perception of sex pheromones [26,27,28,29,30] were not identified. Comparative morphological studies showed that, during the transition to reproductive behavior, the cellular elements of the OE in male yellowfins undergo substantial structural changes. The OSNs demonstrate an enhancement of nuclear–cytoplasmic interactions. This specific arrangement of the mitochondria is not likely to be accidental, reflecting the increased intensity of the energy-dependent transport processes that occur in active OSNs between the nucleus and cytoplasm. Some authors believe that the close contact of mitochondria with the nuclear envelope in metabolically active somatic cells reduces the energy transfer distance to ensure the efficient transport of molecules through the nuclear envelope [55,56]. Moreover, in recent years, it has also been discussed whether the membrane contact sites (MCSs) between mitochondria and nuclear envelope cells are involved not only in energy trafficking but also in the various functional processes in the cell [57]. Thus, the morphological data show that the ultrastructural signs of OSN functional mobilization first develop at the cell body level and then spread to the entire volume of the dendrite (including its terminal), where increased numbers of mitochondria are formed, as well as an ordered network of cytoskeletal elements.

This observation testifies to a heightened level of protein synthesis, which is generally characteristic of nerve cells and their processes during their adaptive functioning [58,59]. During the transition to spawning, the OSN dendrites contain an increased number of mitochondria and orderly arranged microtubules, which can be traced from the perinuclear zone of the cell, ending in the olfactory knob. It should be noted that microtubules have been found in OSN dendrites in many species of fish [60,61,62,63], amphibians [64,65], and mammals [66,67,68]. Despite the large body of data on the structural features of the cytoskeleton in OSNs in various animals, the causal relationship between the formation of microtubules in OSN dendrites and the sensitivity of the latter remains unclear. Are the microtubules able to ensure the transport of ORs and cell signaling system components when the cell is subject to a heightened functional load during the perception of sex pheromones? The ORs belong to the family of G-protein-coupled receptors (GPCRs) [69]. Currently, no evidence is available to suggest that the presentation of ORs in the OSN surface membrane can be mediated by cytoskeletal elements. The anterograde transport of GPCRs, which is mediated by the microtubule network, has been identified only for the Alpha2B-adrenergic receptor [70] and angiotensin II type 1 receptor (AT1R) [71].

It has been shown that the functional expression of most ORs requires the assistance of receptor-transporting proteins (RTPs), but the mechanisms underlying this process remain unclear [72,73,74,75]. It can be assumed that the above-mentioned structural rearrangements in OSNs (including microtubule polymerization) are necessary to increase the OSN’s sensitivity to components of female sex pheromones, but specialized studies are needed to verify this supposition.

During the same period, some of the OSN cells were found to have an activated Golgi apparatus against the background of a metabolically active state, and the cell apex was found to restructure to the mode of dendritic neurosecretion. What is the chemical nature and functional purpose of the secreted product? One of the triggers that lead to the switching of OSNs to dendritic secretion may pertain to the need to detect hydrophobic pheromonal signals from females. Sex pheromones obtained from sexually mature female yellowfin are 17a-hydroxyprogesterone and testosterone [36]. It is known that steroids are one of the predominant pheromones in fish and that they have different solubility in water [76,77].

Some authors [13,78,79] believe that hydrophobic pheromones can also be delivered to receptors by means of small water-soluble odorant-binding proteins (OBPs) from the lipocalin family. OBPs have been described in mammals and *Xenopus*; however, they have not been found in fish. Thus, it can be assumed that dendritic neurosecretion may aim to release either enzymes or OBP-like proteins that increase their solubility in olfactory mucus for subsequent binding to receptors. It is possible that these structural and functional rearrangements in OSNs are due to the hormonal changes typically observed in spawning fish [80,81,82].

Furthermore, one cannot rule out that the dendritic neurosecretion phenomenon may reflect the more generic (unrelated to reproduction) fundamental ability of OSNs to secrete neuropeptides, which can either block odorant molecules of various natures or perform some other functions. Interestingly, the key elements of these rearrangements, i.e., vesicles, have previously been identified in small quantities in dendrites and in the apical regions of cells in fish [60], frogs [64], reptiles [65], rabbits [83], primates [84], humans [85], and other animal species. However, these studies describe no cases of substantial rearrangements of the receptor site in OSNs, which would lead to neurosecretion.

Regardless of the mechanisms that lead to transformation, OSNs that do not contain sensitive cilia probably lose their ability to use chemoreception. Such a course of events may represent one of the mechanisms that predetermine the development of olfactory dysfunction [86,87,88]. Thus, it is currently difficult to fully determine the functional purpose of dendritic neurosecretion. Nevertheless, it can already be stated that the phenomenon of the transformation of OSNs into secretory-type cells indicates the more profound structural and functional plasticity of receptor cells than previously thought. The revealed facts associated with the transformation of OSNs to the dendritic secretion mode are important in the context of dendritic neurosecretion processes that have been vigorously discussed in the last decade and are widely represented in the brain [89,90,91].

In recent years, there has been a growing understanding that SCs largely affect the ability to maintain OSN functional activity and OE regeneration processes [92,93]. SCs enable mechanical [94] and ionic [95,96,97,98] coupling to OSNs. In addition, SCs can secrete various neuromodulators and neurotrophic factors capable of modulating OSN activity [99,100,101,102,103].

The rise in the number of mitochondria in the apical region of ciliated SCs in spawning fish is also of interest. An expanded pool of mitochondria in this local region of the cell may serve as indirect evidence of an increase in ATP production and consumption by the ciliary apparatus of the cells. Model experiments on isolated eukaryotic cells with demembranated flagellum have shown that, at high ATP levels, the rate of contraction and the beat frequency of cilia grow substantially [104]. The increase that we observed in the number of mitochondria in ciliated SCs in spawning fish indicates their increased potential for cilia movement and mucus mixing near OSNs. This may increase the likelihood of the perception of odorous molecules at low concentrations [77,105,106], as in the case of sex pheromones in fish.

## 5. Conclusions

The obtained data show that studying the structural and functional peculiarities of OE cells during the transition from sensitivity to one vital signal (nutritional) to sensitivity to another (pheromonal) allows for an examination of the mechanisms of their adaptation for the optimal perception of a dynamic odorant spectrum in the environment depending on physiological dominance. The principles underlying the increase in male OE sensitivity to female pheromones remain unknown. Is this process connected to an increase in receptor numbers on the cell plasmalemma? Or is OSN (committed to pheromone sensing) neurogenesis needed for this sensitivity increase? This is the first report showing the phenomenon of the transformation of receptor neurons into secretory cells in vertebrates, and it raises questions concerning the mechanisms of this process and its unitarity in the sensory organ in animals of different taxa.

## Data Availability

Data are contained within the article.

## References

[1] Kurian S.M., Naressi R.G., Manoel D., Barwich A.S., Malnic B., Saraiva L.R. (2021). Odor coding in the mammalian olfactory epithelium. Cell Tissue Res..

[2] Francia S., Lodovichi C. (2021). The role of the odorant receptors in the formation of the sensory map. BMC Biol..

[3] Yusuf N., Monahan K. (2024). Epigenetic programming of stochastic olfactory receptor choice. Genesis.

[4] Abbas F., Vinberg F. (2021). Transduction and Adaptation Mechanisms in the Cilium or Microvilli of Photoreceptors and Olfactory Receptors from Insects to Humans. Front. Cell Neurosci..

[5] Pifferi S., Menini A., Kurahashi T., Menini A. (2010). Signal Transduction in Vertebrate Olfactory Cilia. The Neurobiology of Olfaction.

[6] Boccaccio A., Menini A., Pifferi S. (2021). The cyclic AMP signaling pathway in the rodent main olfactory system. Cell Tissue Res..

[7] Tsukahara T., Brann D.H., Pashkovski S., Guitchounts G., Bozza T., Datta S.R. (2021). A transcriptional rheostat couples past activity to future sensory responses. Cell.

[8] Horgue L.F., Assens A., Fodoulian L., Marconi L., Tuberosa J., Haider A., Boillat M., Carleton A., Rodriguez I. (2022). Transcriptional adaptation of olfactory sensory neurons to GPCR identity and activity. Nat. Commun..

[9] Rotermund N., Schulz K., Hirnet D., Lohr C. (2019). Purinergic Signaling in the Vertebrate Olfactory System. Front. Cell Neurosci..

[10] Terral G., Marsicano G., Grandes P., Soria-Gómez E. (2020). Cannabinoid Control of Olfactory Processes: The Where Matters. Genes.

[11] Getchell M.L., Getchell T.V. (1992). Fine structural aspects of secretion and extrinsic innervation in the olfactory mucosa. Microsc. Res. Tech..

[12] Heydel J.M., Coelho A., Thiebaud N., Legendre A., Le Bon A.M., Faure P., Neiers F., Artur Y., Golebiowski J., Briand L. (2013). Odorant-binding proteins and xenobiotic metabolizing enzymes: Implications in olfactory perireceptor events. Anat. Rec..

[13] Pelosi P., Knoll W. (2022). Odorant-binding proteins of mammals. Biol. Rev. Camb. Philos. Soc..

[14] Coppola D.M., Reisert J. (2023). The Role of the Stimulus in Olfactory Plasticity. Brain Sci..

[15] Yamamoto M., Hara T.J. (1982). Comparative morphology of the peripheral olfactory organ in teleosts. Chemoreception in Fishes.

[16] Hansen A., Zielinski B.S. (2005). Diversity in the olfactory epithelium of bony fishes: Development, lamellar arrangement, sensory neuron cell types and transduction components. J. Neurocytol..

[17] Pintos S., Rincon-Camacho L., Pandolfi M., Pozzi A.G. (2020). Morphology and immunohistochemistry of the olfactory organ in the bloodfin tetra, *Aphyocharax anisitsi* (Ostariophysi: Characidae). J. Morphol..

[18] Rincón-Camacho L., Jungblut L.D., Pandolfi M., Pozzi A.G. (2022). Ultrastructural and immunohistochemical characteristics of the olfactory organ cardinal tetra, *Paracheirodon axelrodi* (Characiformes: Characidae). J. Morphol..

[19] Bettini S., Lazzari M., Milani L., Maurizii M.G., Franceschini V. (2023). Immunohistochemical Analysis of Olfactory Sensory Neuron Populations in the Developing Olfactory Organ of the Guppy, *Poecilia reticulata* (Cyprinodontiformes, Poecilidae). Microsc. Microanal..

[20] Speca D.J., Lin D.M., Sorensen P.W. (1999). Functional identification of a goldfish odorant receptor. Neuron.

[21] Hansen A., Rolen S.H., Anderson K., Morita Y., Caprio J., Finger T.E. (2003). Correlation between olfactory receptor cell type and function in the channel catfish. J. Neurosci..

[22] Sato Y., Miyasaka N., Yoshihara Y. (2005). Mutually exclusive glomerular innervation by two distinct types of olfactory sensory neurons revealed in transgenic zebrafish. J. Neurosci..

[23] Thommesen G. (1983). Morphology, distribution, and specificity of olfactory receptor cells in salmonid fishes. Acta Physiol. Scand..

[24] Lipschitz D.L., Michel W.C. (2002). Amino acid odorants stimulate microvillar sensory neurons. Chem. Senses.

[25] Sato K., Suzuki N. (2001). Whole-cell response characteristics of ciliated and microvillous olfactory receptor neurons to amino acids, pheromone candidates and urine in rainbow trout. Chem. Senses.

[26] Hansen A., Finger T.E. (2000). Phyletic distribution of crypt-type olfactory receptor neurons in fishes. Brain Behav. Evol..

[27] Ahuja G., Nia S., Zapilko V., Shiriagin V., Kowatschew D., Oka Y., Korsching S.I. (2014). Kappe neurons, a novel population of olfactory sensory neurons. Sci. Rep..

[28] Hamdani E.H., Døving K.B. (2007). The functional organization of the fish olfactory system. Prog. Neurobiol..

[29] Vielma A., Ardiles A., Delgado L., Schmachtenberg O. (2008). The elusive crypt olfactory receptor neuron: Evidence for its stimulation by amino acids and cAMP pathway agonists. J. Exp. Biol..

[30] Bazáes A., Schmachtenberg O. (2012). Odorant tuning of olfactory crypt cells from juvenile and adult rainbow trout. J. Exp. Biol..

[31] Braubach O.R., Fine A., Croll R.P. (2012). Distribution and functional organization of glomeruli in the olfactory bulbs of zebrafish (*Danio rerio*). J. Comp. Neurol..

[32] Wakisaka N., Miyasaka N., Koide T., Masuda M., Hiraki-Kajiyama T., Yoshihara Y. (2017). An Adenosine Receptor for Olfaction in Fish. Curr. Biol..

[33] Dieris M., Kowatschew D., Korsching S.I. (2021). Olfactory function in the trace amine-associated receptor family (TAARs) evolved twice independently. Sci. Rep..

[34] Dmitrieva T.M., Ostroumov V.A., Galazy G.I. (1987). Role of chemical communication in reproductive behaviour of yellowfin Baikal sculpin. Morphology and Ecology of Fish.

[35] Katsel P.L., Dmitrieva T.M., Valeyev R.B., Kozlov Y.P. (1992). Sex pheromones of male yellowfin Baikal sculpin (*Cottocomephorus grewingkii*): Isolation and chemical studies. J. Chem. Ecol..

[36] Valeeva N.I. (1997). Ecological and Biochemical Interactions of Sculpins (Cottoidei) of Lake Baikal During the Reproductive Period. Ph.D. Thesis.

[37] Taliev D.N. (1955). Lake Baikal Sculpins (Cottoidei).

[38] Sideleva V.G. (2003). Endemic Fishes of Lake Baikal.

[39] Sideleva V.G., Kozlova T.A. (2010). The comparative study of endemic cottoid fishes (Cottidae, Comephoridae) and their adaptation to pelagic habitat in Lake Baikal. Proc. Zool. Inst. RAS.

[40] Dadzie S., Wangila B. (1980). Reproductive biology, length-weight relationship and relative condition of pondraised Tilapia zilli (Gervais). J. Fish. Biol..

[41] Macirella R., Madeo G., Sesti S., Tripepi M., Bernabò I., Godbert N., La Russa D., Brunelli E. (2020). Exposure and post-exposure effects of chlorpyrifos on Carassius auratus gills: An ultrastructural and morphofunctional investigation. Chemosphere.

[42] Reynolds E.S. (1963). The use of lead citrate at high pH as an electron-opaque stain in electron microscopy. J. Cell Biol..

[43] Sudakov N.P., Chang H.M., Renn T.Y., Klimenkov I.V. (2023). Degenerative and Regenerative Actin Cytoskeleton Rearrangements, Cell Death, and Paradoxical Proliferation in the Gills of Pearl Gourami (*Trichogaster leerii*) Exposed to Suspended Soot Microparticles. Int. J. Mol. Sci..

[44] Klimenkov I.V., Sudakov N.P., Pastukhov M.V., Kositsyn N.S. (2020). The Phenomenon of Compensatory Cell Proliferation in Olfactory Epithelium in Fish Caused by Prolonged Exposure to Natural Odorants. Sci. Rep..

[45] Burton P.R. (1992). Ultrastructural studies of microtubules and microtubule organizing centers of the vertebrate olfactory neuron. Microsc. Res. Tech..

[46] Ching K., Wang J.T., Stearns T. (2022). Long-range migration of centrioles to the apical surface of the olfactory epithelium. Elife.

[47] Klimenkov I.V., Sudakov N.P., Pastukhov M.V., Kositsyn N.S. (2016). Cytochemical features of olfactory receptor cells in benthic and pelagic sculpins (Cottoidei) from Lake Baikal. Arch. Biol. Sci..

[48] Nozumi M., Igarashi M. (2018). Vesicular movements in the growth cone. Neurochem. Int..

[49] Leite S.C., Pinto-Costa R., Sousa M.M. (2021). Actin dynamics in the growth cone: A key player in axon regeneration. Curr. Opin. Neurobiol..

[50] Graziadei P.P., Graziadei G.A. (1979). Neurogenesis and neuron regeneration in the olfactory system of mammalsI. Morphological aspects of differentiation and structural organization of the olfactory sensory neurons. J. Neurocytol..

[51] Hentig J.T., Byrd-Jacobs C.A. (2016). Exposure to Zinc Sulfate Results in Differential Effects on Olfactory Sensory Neuron Subtypes in Adult Zebrafish. Int. J. Mol. Sci..

[52] Xie C., Habif J., Uytingco C.R., Ukhanov K., Zhang L., de Celis C., Sheffield V.C., Martens J.R. (2021). Gene therapy rescues olfactory perception in a clinically relevant ciliopathy model of Bardet-Biedl syndrome. FASEB J..

[53] Silflow C.D., Lefebvre P.A. (2001). Assembly and motility of eukaryotic cilia and flagella. Lessons from *Chlamydomonas reinhardtii*. Plant Physiol..

[54] Klimenkov I.V., Sudakov N.P., Pastukhov M.V., Svinov M.M., Kositsyn N.S. (2018). Rearrangement of Actin Microfilaments in the Development of Olfactory Receptor Cells in Fish. Sci. Rep..

[55] Dzeja P.P., Bortolon R., Perez-Terzic C., Holmuhamedov E.L., Terzic A. (2002). Energetic communication between mitochondria and nucleus directed by catalyzed phosphotransfer. Proc. Natl. Acad. Sci. USA.

[56] Prachar J. (2003). Intimate contacts of mitochondria with nuclear envelope as a potential energy gateway for nucleo-cytoplasmic mRNA transport. General. Physiol. Biophys..

[57] Ovciarikova J., Shikha S., Sheiner L. (2002). Nuclear Interactions: A Spotlight on Nuclear Mitochondrial Membrane Contact Sites. Contact.

[58] Holt C.E., Martin K.C., Schuman E.M. (2019). Local translation in neurons: Visualization and function. Nat. Struct. Mol. Biol..

[59] Dastidar S.G., Nair D.A. (2022). Ribosomal Perspective on Neuronal Local Protein Synthesis. Front. Mol. Neurosci..

[60] Bannister L.H. (1965). The Fine Structure of the Olfactory Surface of Teleostean Fishes. Q. J. Microsc. Sci..

[61] Theisen B. (1972). Ultrastructure of the olfactory epithelium in the Australian lungfish Neoceratodus forsteri. Acta Zool..

[62] Datta N.C., Bandopadhyay S. (1997). Ultrastructure of cell types of the olfactory epithelium in a catfish, Heteropneustesfossilis (Bloch). J. Biosci..

[63] Ghosh S.K. (2021). The olfactory organ of schilbid catfish *Eutropiichthys vacha* (Hamilton, 1822): Morphological and ultrastructural studies. JoBAZ.

[64] Burton P.R. (1985). Ultrastructure of the olfactory neuron of the bullfrog: The dendrite and its microtubules. J. Comp. Neurol..

[65] Hansen A. (2007). Olfactory and solitary chemosensory cells: Two different chemosensory systems in the nasal cavity of the American alligator, Alligator mississippiensis. BMC Neurosci..

[66] Cuschieri A., Bannister L.H. (1975). The development of the olfactory mucosa in the mouse: Electron microscopy. J. Anat..

[67] Jafek B.W., Murrow B., Michaels R., Restrepo D., Linschotenm M. (2002). Biopsies of human olfactory epithelium. Chem. Senses.

[68] Sahin E., Ortug G., Ortug A. (2018). Does cigarette smoke exposure lead to histopathological alterations in the olfactory epithelium? An electron microscopic study on a rat model. Ultrastruct. Pathol..

[69] Buck L., Axel R. (1991). A novel multigene family may encode odorant receptors: A molecular basis for odor recognition. Cell.

[70] Duvernay M.T., Wang H., Dong C., Guidry J.J., Sackett D.L., Wu G. (2011). Alpha2B-adrenergic receptor interaction with tubulin controls its transport from the endoplasmic reticulum to the cell surface. J. Biol. Chem..

[71] Zhang X., Wang H., Duvernay M.T., Zhu S., Wu G. (2013). The angiotensin II type 1 receptor C-terminal Lys residues interact with tubulin and modulate receptor export trafficking. PLoS ONE.

[72] Bush C.F., Hall R.A. (2008). Olfactory receptor trafficking to the plasma membrane. Cell Mol. Life Sci..

[73] Sharma R., Ishimaru Y., Davison I., Ikegami K., Chien M.S., You H., Chi Q., Kubota M., Yohda M., Ehlers M. (2017). Olfactory receptor accessory proteins play crucial roles in receptor function and gene choice. Elife.

[74] Fukutani Y., Tamaki R., Inoue R., Koshizawa T., Sakashita S., Ikegami K., Ohsawa I., Matsunami H., Yohda M. (2019). The N-terminal region of RTP1S plays important roles in dimer formation and odorant receptor-trafficking. J. Biol. Chem..

[75] Inoue R., Fukutani Y., Niwa T., Matsunami H., Yohda M. (2023). Identification and Characterization of Proteins That Are Involved in RTP1S-Dependent Transport of Olfactory Receptors. Int. J. Mol. Sci..

[76] Stewart M., Baker C.F., Sorensen P.W. (2013). Chemical analysis of aquatic pheromones in fish. Methods Mol. Biol..

[77] Bowers J.M., Li C.Y., Parker C.G., Westbrook M.E., Juntti S.A. (2023). Pheromone Perception in Fish: Mechanisms and Modulation by Internal Status. Integr. Comp. Biol..

[78] Sun J.S., Xiao S., Carlson J.R. (2018). The diverse small proteins called odorant-binding proteins. Open Biol..

[79] Zaremska V., Renzone G., Arena S., Ciaravolo V., Buberl A., Balfanz F., Scaloni A., Knoll W., Pelosi P. (2022). An odorant-binding protein in the elephant’s trunk is finely tuned to sex pheromone (Z)-7-dodecenyl acetate. Sci. Rep..

[80] Sokołowska E., Kleszczyńska A., Nietrzeba M., Kulczykowska E. (2015). Annual changes in brain concentration of arginine vasotocin and isotocin correspond with phases of reproductive cycle in round goby, *Neogobius melanostomus*. Chronobiol. Int..

[81] Mennigen J.A., Ramachandran D., Shaw K., Chaube R., Joy K.P., Trudeau V.L. (2022). Reproductive roles of the vasopressin/oxytocin neuropeptide family in teleost fishes. Front. Endocrinol..

[82] Zohar Y. (2021). Fish reproductive biology—Reflecting on five decades of fundamental and translational research. Gen. Comp. Endocrinol..

[83] de Lorenzo A.J. (1957). Electron microscopic observations of the olfactory mucosa and olfactory nerve. J. Biophys. Biochem. Cytol..

[84] Loo S.K. (1977). Fine structure of the olfactory epithelium in some primates. J. Anat..

[85] Polyzonis B.M., Kafandaris P.M., Gigis P.I., Demetriou T. (1979). An electron microscopic study of human olfactory mucosa. J. Anat..

[86] Marin C., Vilas D., Langdon C., Alobid I., López-Chacón M., Haehner A., Hummel T., Mullol J. (2018). Olfactory Dysfunction in Neurodegenerative Diseases. Curr. Allergy Asthma Rep..

[87] Prediger R.D., Schamne M.G., Sampaio T.B., Moreira E.L.G., Rial D. (2019). Animal models of olfactory dysfunction in neurodegenerative diseases. Handb. Clin. Neurol..

[88] Doty R.L., Hawkes C.H. (2019). Chemosensory dysfunction in neurodegenerative diseases. Handb. Clin. Neurol..

[89] Ovsepian S.V., Dolly J.O. (2011). Dendritic SNAREs add a new twist to the old neuron theory. Proc. Natl. Acad. Sci. USA.

[90] Ludwig M., Apps D., Menzies J., Patel J.C., Rice M.E. (2016). Dendritic Release of Neurotransmitters. Compr. Physiol..

[91] Kulik Y.D., Watson D.J., Cao G., Kuwajima M., Harris K.M. (2019). Structural plasticity of dendritic secretory compartments during LTP-induced synaptogenesis. Elife.

[92] Frontera J.L., Cervino A.S., Jungblut L.D., Paz D.A. (2015). Brain-derived neurotrophic factor (BDNF) expression in normal and regenerating olfactory epithelium of Xenopus laevis. Ann. Anat..

[93] Bryche B., Baly C., Meunier N. (2021). Modulation of olfactory signal detection in the olfactory epithelium: Focus on the internal and external environment, and the emerging role of the immune system. Cell Tissue Res..

[94] Liang F. (2020). Sustentacular Cell Enwrapment of Olfactory Receptor Neuronal Dendrites: An Update. Genes.

[95] Vogalis F., Hegg C.C., Lucero M.T. (2005). Ionic conductances in susten-tacular cells of the mouse olfactory epithelium. J. Physiol..

[96] Hegg C.C., Irwin M., Lucero M.T. (2009). Calcium store-mediated signaling in sustentacular cells of the mouse olfactory epithelium. Glia.

[97] Zhang C. (2010). Gap junctions in olfactory neurons modulate olfactory sensitivity. BMC Neurosci..

[98] Henriques T., Agostinelli E., Hernandez-Clavijo A., Maurya D., Rock J., Harfe B.D., Menini A., Pifferi S. (2019). TMEM16A calcium-activated chloride currents in supporting cells of the mouse olfactory epithelium. J. Gen. Physiol..

[99] Hansel D.E., Eipper B.A., Ronnett G.V. (2001). Neuropeptide Y functions as a neuroproliferative factor. Nature.

[100] Czesnik D., Schild D., Kuduz J., Manzini I. (2007). Cannabinoid action in the olfactory epithelium. Proc. Natl. Acad. Sci. USA.

[101] Breunig E., Manzini I., Piscitelli F., Gutermann B., Di Marzo V., Schild D., Czesnik D. (2010). The endocannabinoid 2-arachidonoyl-glycerol controls odor sensitivity in larvae of Xenopus laevis. J. Neurosci..

[102] Hayoz S., Jia C., Hegg C. (2012). Mechanisms of constitutive and ATP-evoked ATP release in neonatal mouse olfactory epithelium. BMC Neurosci..

[103] Acevedo C., Blanchard K., Bacigalupo J., Vergara C. (2019). Possible ATP trafficking by ATP-shuttles in the olfactory cilia and glucose transfer across the olfactory mucosa. FEBS Lett..

[104] Chen D.T.N., Heymann M., Fraden S., Nicastro D., Dogic Z. (2015). ATP Consumption of Eukaryotic Flagella Measured at a Single-Cell Level. Biophys. J..

[105] Stacey N., Sorensen P. (2009). Hormonal Pheromones in Fish. Horm. Brain Behavior..

[106] Sorensen P.W., Levesque H.M. (2021). Hormonal Prostaglandin F2α Mediates Behavioral Responsiveness to a Species-Specific Multi-component Male Hormonal Sex Pheromone in a Female Fish. Integr. Comp. Biol..

